# Orexin/Hypocretin and MCH Neurons: Cognitive and Motor Roles Beyond Arousal

**DOI:** 10.3389/fnins.2021.639313

**Published:** 2021-03-22

**Authors:** Cristina Concetti, Denis Burdakov

**Affiliations:** Department of Health Sciences and Technology, ETH Zürich, Zurich, Switzerland

**Keywords:** hypothalamus, neuropeptide, orexin, melanin-concentrating hormone, memory, locomotion

## Abstract

The lateral hypothalamus (LH) is classically implicated in sleep-wake control. It is the main source of orexin/hypocretin and melanin-concentrating hormone (MCH) neuropeptides in the brain, which have been both implicated in arousal state switching. These neuropeptides are produced by non-overlapping LH neurons, which both project widely throughout the brain, where release of orexin and MCH activates specific postsynaptic G-protein-coupled receptors. Optogenetic manipulations of orexin and MCH neurons during sleep indicate that they promote awakening and REM sleep, respectively. However, recordings from orexin and MCH neurons in awake, moving animals suggest that they also act outside sleep/wake switching. Here, we review recent studies showing that both orexin and MCH neurons can rapidly (sub-second-timescale) change their firing when awake animals experience external stimuli, or during self-paced exploration of objects and places. However, the sensory-behavioral correlates of orexin and MCH neural activation can be quite different. Orexin neurons are generally more dynamic, with about 2/3rds of them activated before and during self-initiated running, and most activated by sensory stimulation across sensory modalities. MCH neurons are activated in a more select manner, for example upon self-paced investigation of novel objects and by certain other novel stimuli. We discuss optogenetic and chemogenetic manipulations of orexin and MCH neurons, which combined with pharmacological blockade of orexin and MCH receptors, imply that these rapid LH dynamics shape fundamental cognitive and motor processes due to orexin and MCH neuropeptide actions in the awake brain. Finally, we contemplate whether the awake control of psychomotor brain functions by orexin and MCH are distinct from their “arousal” effects.

## A Brief Historical Overview and Introduction

Based on behavioral effects of anatomically targeted lesions, the lateral hypothalamus (LH) has been long recognized as a key brain center in the control of appetite and arousal [reviewed in Saper et al. (2005); Burdakov et al. (2013), Stuber and Wise (2016); Herrera et al. (2017), Bracey and Burdakov (2020)]. It is now known to contain neurochemically and biophysically heterogeneous neuronal populations, which express a plethora of neuropeptides and almost invariably co-express the classic fast neurotransmitters, GABA and glutamate (Schöne et al., 2011; Schöne and Burdakov, 2012; Stuber and Wise, 2016; Romanov et al., 2017; Kosse and Burdakov, 2018; Mickelsen et al., 2019). Some of these neuropeptides, such as orexin/hypocretin and melanin-concentrating hormone (MCH) are generally thought to be unique to the LH (i.e., they are not made anywhere else in the brain) (de Lecea et al., 1998; Sakurai et al., 1998; Bittencourt, 2011). Although the canonical appetite and arousal roles of LH neurons have been receiving the most attention, there is also historic and modern evidence that can be interpreted to place the LH among essential brain structures in cognitive and motor control (Marshall et al., 1971; Schwartz and Teitelbaum, 1974; Levitt and Teitelbaum, 1975; Karnani et al., 2020).

This article presents recent experimental evidence and interpretations linking these “non-canonical” LH functions to specific neuropeptidergic neural subsets of the LH, specifically orexin and MCH neurons. The overall aim of this review is to place a small number of recent studies from the authors’ laboratory in a broader context; for more exhaustive reviews of the LH the readers are referred elsewhere (e.g., Saper et al., 2005; Burdakov et al., 2013; Stuber and Wise, 2016; Bracey and Burdakov, 2020). First, we will review basic physiological and anatomical properties of these neurons at the cellular and molecular levels. Second, we will highlight some recent studies linking temporally defined, brief activity epochs of these neurons in the awake brain to specific aspects of behavior and cognition. Finally, we will highlight the many remaining questions, and present some arguments that orexin and MCH neurons dynamically shape brain function beyond their canonical roles in “arousal.”

## Orexin and MCH Cells Control Their Neural Targets via Glutamate/Gaba and Peptide Co-transmission

Orexin neurons are known to be essential for stable wakefulness, and their loss produces the sleep disorder narcolepsy across mammalian species (Chemelli et al., 1999; Lin et al., 1999; Nishino et al., 2000; Thannickal et al., 2000; Bassetti et al., 2019). MCH neurons have been reported to promote REM sleep (Verret et al., 2003; Jego et al., 2013), though some studies also conclude that they can promote NREM sleep (Konadhode et al., 2013). These sleep/arousal effects of orexin and MCH neurons will not be discussed here, since they have been the subject of many recent publications (Ferreira et al., 2017; Bassetti et al., 2019; Burdakov, 2019; Adamantidis et al., 2020).

Orexin and MCH immunoreactivities do not overlap, implying that these peptides are made by distinct classes of LH neurons (Broberger et al., 1998; Peyron et al., 1998; Mickelsen et al., 2017). The two cell types are also thought to have opposing roles on arousal, and correspondingly often show reciprocal activity profiles *in vivo* and in brain slices, and are differentially modulated by some indicators of body energy status, such as glucose (Hassani et al., 2009; Karnani and Burdakov, 2011; Apergis-Schoute et al., 2015; Kosse et al., 2015; Burdakov and Adamantidis, 2020). However, in many other ways, the two cell types are similar. They both project their axons widely throughout the brain, with innervations not only of regions regulating arousal and reward, but also many other aspects of cognition and motor control (Peyron et al., 1998; Bittencourt, 2011; Gonzalez et al., 2012; Jego et al., 2013). Both cell types also receive brain-wide monosynaptic innervations (Gonzalez et al., 2016a). The specific G-protein coupled receptors (GPCRs) for orexin and MCH are expressed equally widely in the brain, with only some differences, and the distribution of the receptors generally (but not always) mirrors that of projections (Saito et al., 2001; Sakurai, 2007). Orexin binding to orexin GPCRs is rapidly coupled to depolarization and excitation of neuronal plasma membranes, due to activation of non-selective cation channels and/or Na^+^/Ca^2+^ exchangers (Eriksson et al., 2001; Burdakov et al., 2003; Burdakov, 2004; Kukkonen and Leonard, 2014). MCH binding to MCH GPCR has less clear electrical effects, but has been linked to control of glutamate receptor expression and function, or to control of presynaptic transmitter release (Gao and van den Pol, 2001; Wu et al., 2009; Pachoud et al., 2010).

In addition to orexin and MCH peptides, both neural types express the classic “fast” neurotransmitters. For orexin neurons, many histological and functional studies agree that their fast co-transmitter is mostly glutamate (Schöne and Burdakov, 2012). Indeed, when orexin neurons are optogenetically stimulated, the postsynaptic electrical excitation involves both glutamatergic and orexinergic components, which can be pharmacologically dissociated at both intrahypothalamic (Schöne et al., 2012, 2014) and extrahypothalamic (Sears et al., 2013; Blomeley et al., 2018) projection targets. Orexin receptor -mediated postsynaptic excitation gradually “ramps up” during steady-rate orexin neuron stimulation, and this ramping has been proposed to be an outcome of temporal integration of presynaptic input, which – in the context of feedback loops of which orexin neurons are part – reveals a potential computational mechanism (formally known as integral feedback control) for wakefulness stability implemented by the orexin system (Kosse and Burdakov, 2014; Schöne et al., 2014; Schone and Burdakov, 2017).

For MCH neurons, the nature of their fast co-transmitter is more controversial. Molecular screens identify markers for both GABA and glutamate in MCH cells (Del Cid-Pellitero and Jones, 2012; Chee et al., 2015). Optogenetic stimulation of MCH cells produces GABAergic outputs inside the hypothalamus, but glutamatergic outputs in other brain areas (Jego et al., 2013; Chee et al., 2015). The possibility remains that there may be distinct subsets of GABAergic and glutamatergic MCH cells is being explored, but there is not yet a clear logic for how this is arranged in the brain. Overall, despite clear evidence for intra- and extrahypothalamic functional neural circuits made by orexin and MCH neurons (Apergis-Schoute et al., 2015; Kosse et al., 2017; Kosse and Burdakov, 2019; Adamantidis et al., 2020; Burdakov, 2020), at the multiple projection targets of orexin and MCH neurons in the brain, the relative roles of their peptide and small-molecule neurotransmitters remain incompletely understood overall.

## Orexin Neuron Dynamics Underlying Sensorimotor Control and Spatial Exploration

Brain slice patch clamp recordings from orexin neurons show that they can intrinsically generate tonic firing in a regular, pacemaker-like manner (van den Pol et al., 1998; Li et al., 2002; Eggermann et al., 2003; Yamanaka et al., 2003b; Burdakov et al., 2004, 2005; Williams et al., 2007, 2011; Gonzalez et al., 2009b; Williams and Burdakov, 2009; Schöne et al., 2011). *In vitro*, this intrinsic activity can be slowly modulated by specific nutrients, gasses, and neuromodulators (Li et al., 2002; Yamanaka et al., 2003a; Williams et al., 2007; Gonzalez et al., 2008, 2009a, c; Karnani et al., 2011a, b; Carus-Cadavieco et al., 2017). However, the activity dynamics of orexin neurons *in vivo* change much more rapidly than in brain slices, likely reflecting the brain-wide neural inputs that they receive (Gonzalez et al., 2016a). Orexin cell activity of awake rodents responds to sensory stimuli on a subsecond timescale, and this activation correlates with muscle/EMG activation and movement (Lee et al., 2005; Mileykovskiy et al., 2005; Takahashi et al., 2008; Gonzalez et al., 2016a, b; Hassani et al., 2016; Burdakov and Peleg-Raibstein, 2019). In this section, we review some emerging roles of this awake orexin cell activity, focusing on a small selection of recent studies.

**FIGURE 1:**
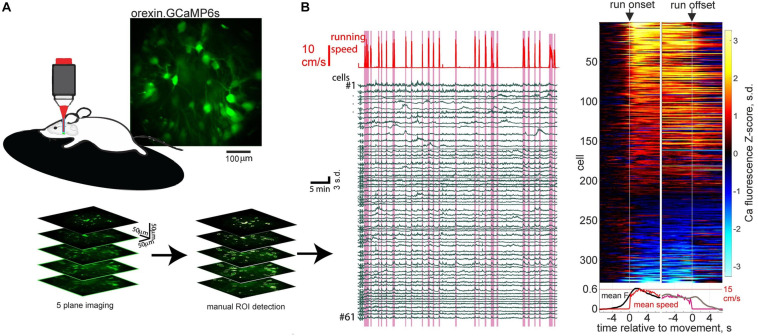
Orexin neural ensemble dynamics underlying running initiation. **(A)** 2-photon volumetric imaging of multiple orexin neurons in head-fixed mice running on a treadmill. **(B)** Orexin cell activity profiles aligned to onset and offset of self-initiated running bouts. Source: Karnani et al. (2020).

Recent orexin neural network imaging at cellular resolution indicates that the rapid dynamics of orexin cells during wakefulness appears to be a property of most orexin cells. 2-photon calcium imaging of >300 orexin neurons during locomotion reveals that the majority (around 70%) of orexin neurons activate around initiation of running bouts (Karnani et al., 2020; Figure 1). Optogenetic evidence indicates that this peri-initiation activity of orexin cells appears to be causally linked to locomotion initiation. Optogenetic excitation of orexin cells at frequencies resembling their natural *in vivo* firing (Lee et al., 2005; Mileykovskiy et al., 2005), produces frequency-dependent running (Karnani et al., 2020). In turn, optogenetic inhibition of orexin neurons makes both sensory-evoked and self-paced running less likely (Karnani et al., 2020).

These experiments supply causal evidence for a role of orexin neurons in rapid sensorimotor control in the awake brain (Karnani et al., 2020). This augments the previous conceptualization of orexin neurons as slow modulators of sleep states. While previously orexin neurons were known to display rapid sensory responses *in vivo* (Mileykovskiy et al., 2005; Gonzalez et al., 2016a, b; Hassani et al., 2016), until the study of Karnani et al. (2020), the causal role of these rapid activity changes was unknown. The finding that subsecond sensory dynamics of orexin cells produces rapid locomotor control, which is not entirely dissimilar to cortical-mediated sensorimotor transformations (Ferezou et al., 2007; Svoboda and Li, 2018), clarifies why orexin cells may need to update their awake activity on a subsecond timescale. However, the downstream mechanisms underlying the fast sensorimotor control by orexin cells, such as the postsynaptic CNS regions responsible and the roles of co-released orexin vs. glutamate, remain unknown at present.

**FIGURE 2:**
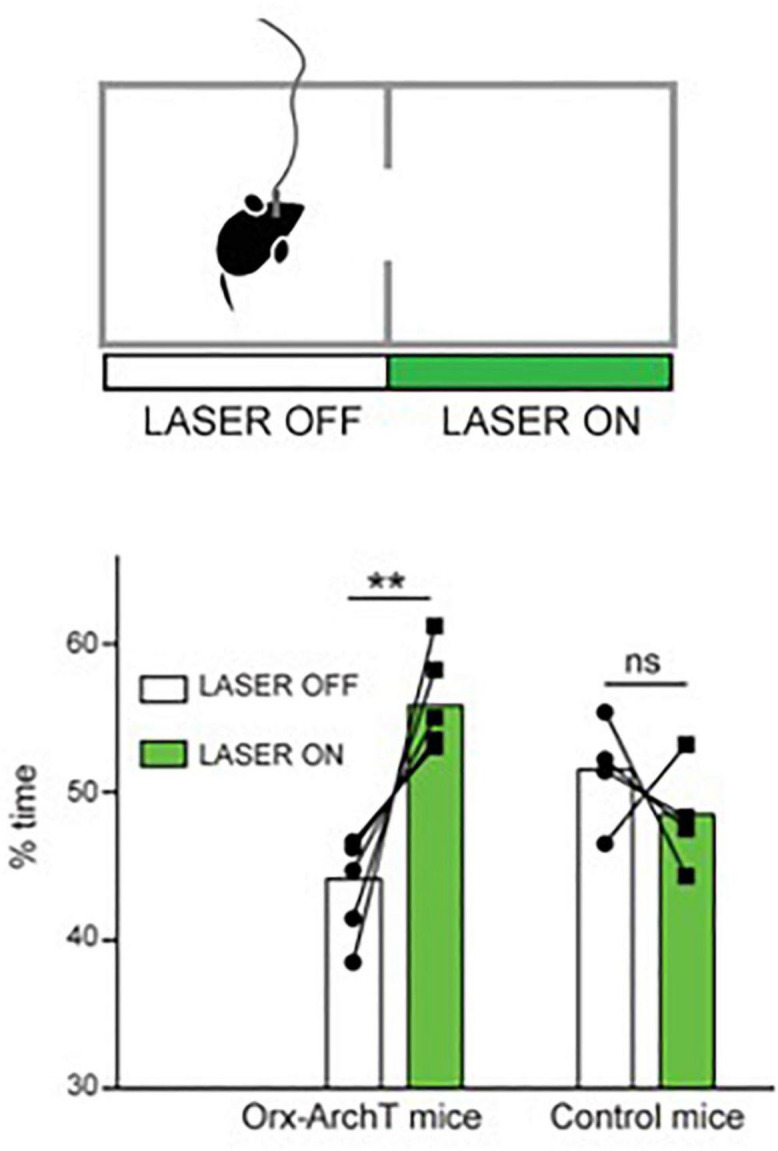
Role of natural orexin cell activity in spatial avoidance. Optogenetic silencing (“LASER ON”) of orexin-ArchT neuron in one half of exploration arena causes mice to spend more time in that half. Source: Garau et al. (2020).

Another recent study examined the role of the awake activity of orexin neurons by optogenetic silencing of this activity during self-paced spatial exploration in awake mice (Garau et al., 2020). Optosilencing of orexin cells caused mice to spend more time in the silencing-associated place in real-time place preference tests, where the cells are optosilenced in video-controlled closed-loop based on the spatial position of the animal in the experimental arena (Garau et al., 2020; Figure 2). In turn, constant orexin cell silencing in arenas containing regions that are aversive for mice (exposed or bright-illuminated regions) caused mice to increase their self-paced entries into these aversive regions (Garau et al., 2020). It is important to note that these are short-lasing experiments, typically taking a few minutes, during which the mouse is actively exploring the cage and does not enter sleep (at least not the kind of sleep where the animal cannot walk). Together with similar studies involving optogenetic stimulation of orexin cells (Giardino et al., 2018), this suggest that “awake” orexin cell activity may create an aversive brain state that drives the animal to move while avoiding potentially dangerous places (Burdakov, 2019, 2020; Garau et al., 2020). We note that viewing the orexin system as a “stress/aversion” system is, in our opinion, not contradictory to viewing it as a “reward-seeking” system (Harris et al., 2005; Harris and Aston-Jones, 2006), because reward-seeking is often driven by reward-deficit (i.e., stress), and so a “stress” neural signal would be expected to drive reward seeking [this point has also been made well by others, notably (Boutrel et al., 2005)].

Optogenetics-assisted circuit mapping and combinatorial chemogenetic and pharmacological experiments *in vivo* suggest that downstream, such actions of orexin neurons may involve orexin projections to action-selection control regions such as the nucleus accumbens (Blomeley et al., 2018); and upstream – inputs from food-seeking control neurons such as hypothalamic Agrp neurons (Garau et al., 2020). The extent to which orexin neuropeptide vs. coreleased glutamate contributes to this is not entirely clear. However, optogenetic stimulation of orexin cells suggests that, in D2 neurons of accumbens shell, the postsynaptic excitation induced by orexin axon firing is mediated predominantly by orexin rather than glutamate transmission (Blomeley et al., 2018). Furthermore, local infusion of orexin peptide into the accumbens shell triggers increased place aversion consistent with the role of accumbal orexin actions in this process (Blomeley et al., 2018). Considering that there is evidence from orexin cell recordings that orexin neurons activity increases when the animal finds itself in a potentially aversive place (Garau et al., 2020), orexin cells may thus be a part of behavioral control loop that creates optimal brain states for increased locomotion and reduced risk-taking in response to sensory evidence. Interestingly, the same or overlapping dopaminergic circuits have been also proposed to play a role in sleep/wake control (Lazarus et al., 2013; Eban-Rothschild et al., 2016; Chowdhury et al., 2019; Yu et al., 2019).

## Mch Neuron Dynamics Underlying Awake Experiences and Subsequent Memories

Based on pioneering electrophysiological recordings from a small number of MCH neurons in head-fixed rats (Hassani et al., 2009), for some time it was thought that these cells are only active during sleep. These activity of MCH cells during sleep was subsequently linked to sleep-state switching (Jego et al., 2013), and more recently to memory regulation during REM sleep (Izawa et al., 2019). However, in 2016, it was discovered through fiber photometry recordings of MCH cell populations that these cells are also active during awake behavior (Gonzalez et al., 2016a), in line with previous reports of sensitization of locomotion to psychoactive drugs in MCH or MCH receptor deficient mice (Smith et al., 2005; Tyhon et al., 2006; Pissios et al., 2008; Whiddon and Palmiter, 2013; Chee et al., 2019). Mouse MCH neurons appeared to generate large activity bursts during self-paced awake exploration of novel objects (Gonzalez et al., 2016a; Blanco-Centurion et al., 2019). In this section, we review two studies focusing on elucidation of the function of these awake, context-specific waves of MCH neuronal activity, carried out using temporally targeted reversible optosilencing of this activity in behaving mice.

**FIGURE 3:**
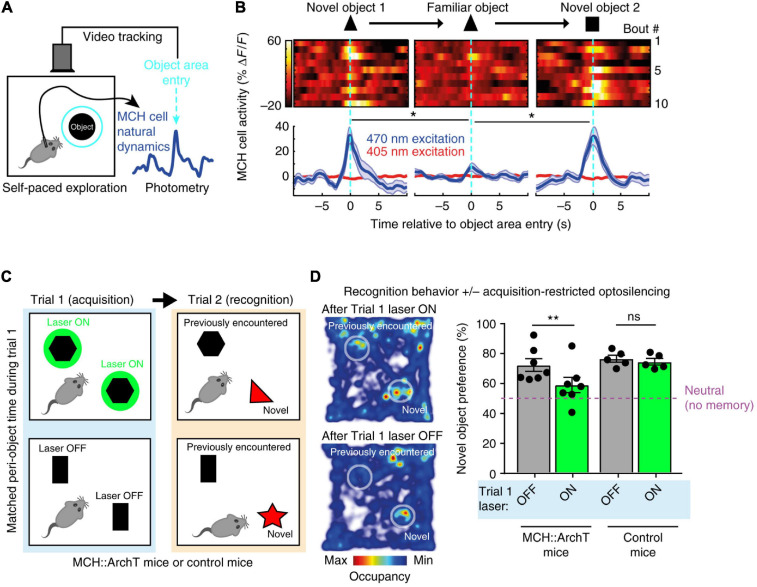
Role of exploration-associated MCH cell activity in object recognition memory. **(A)** Schematic of MCH-GCaMP recording aligned to self-paced object exploration. **(B)** MCH cell activity associated with exploration of novel (but not familiar) objects. **(C)** Schematic of MCH-ArchT cell silencing selectivity during self-paced object exploration. **(D)** No object recognition after MCH cells were silenced upon the earlier object exploration. Source: Kosse and Burdakov (2019).

The first study carried out video-controlled closed-loop optogenetic silencing of MCH cell activity waves associated with self-paced exploration of novel objects (Kosse and Burdakov, 2019). Importantly, large MCH activity waves were associated with exploration of novel but not familiar objects, consistent with novelty representations (Kosse and Burdakov, 2019; Figures 3A,B). The MCH cell optogenetic silencing, selectively during the moments of object memory encoding, disrupted later recognition of the encountered objects, suggesting that MCH cell activity during novel object exploration is required for formation and later expression of object recognition memory (Kosse and Burdakov, 2019; Figures 3C,D). Channelrhodopsin-assisted circuit mapping in brain slices, and chemogenetic and pharmacological experiments *in vivo*, revealed that MCH neurons are under inhibitory GABAergic control by local LH GAD65 neurons (Kosse and Burdakov, 2019). Optogenetic silencing of the LH GAD65 neurons during novel object investigations augmented subsequent object recognition, and this augmentation was blocked by MCH receptor antagonist (Kosse and Burdakov, 2019).

These findings suggest that mice fail to recognize objects unless their MCH cell activity “marks” prior encounters with the objects, and that a GAD65→MCH LH circuit shapes the size of the memory-formation-gating MCH cell activity. The object recognition memory investigated in this study is important for normal life of mammals (Winters et al., 2008; Broadbent et al., 2010). Earlier molecular and pharmacological studies linked MCH neuropeptide action to avoidance memory (Adamantidis and de Lecea, 2009; Pachoud et al., 2010) but contained no information about when MCH cell activity influences memory, nor how memory-gating MCH signals are controlled at the circuit level. Kosse et al., thus supplied causal evidence for the role of awake, object-exploration-associated MCH cell activity signals in object recognition memory formation. Importantly – considering earlier reports linking MCH cell deficiency to hyperlocomotion (Shimada et al., 1998; Whiddon and Palmiter, 2013) which may affect novel object exploration time – the study of Kosse et al., monitored and controlled the total time that mice spent with novel object, indicating that the effects of MCH cell optogenetic manipulations could not be explained simply by changes in the duration of sensory exposure to the object.

**FIGURE 4:**
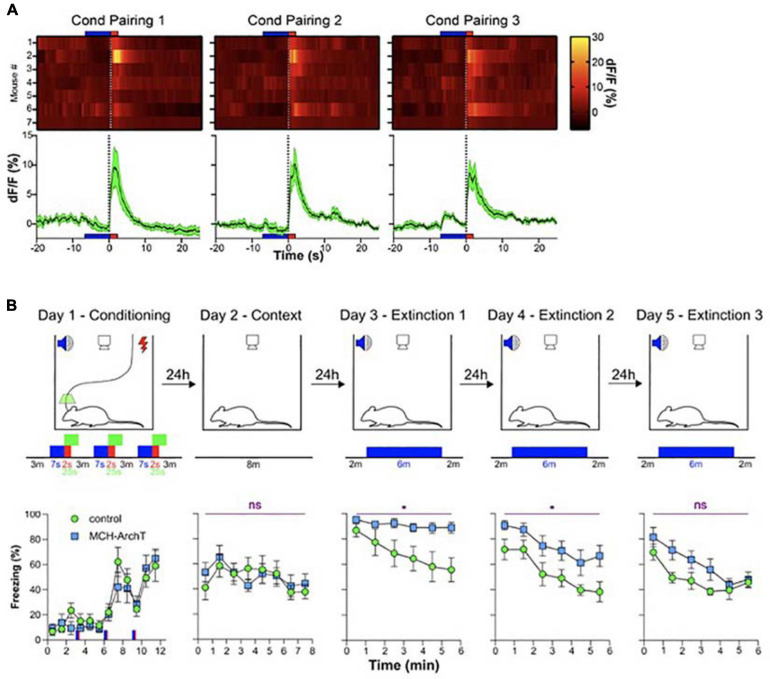
Role of MCH neuron activity during fear conditioning in cued fear extinction. **(A)** MCH-GCaMP population dynamics during auditory fear conditioning (blue, tone; red, footshock). **(B)** Effect of MCH-GCaMP cell silencing during conditioning (green squares) on subsequent cued fear extinction, Top row, schematic of experiment; Bottom row, corresponding fear behavior. Source: Concetti et al. (2020). *p < 0.05; ns, *p*≥0.05.

The second study probing the function of MCH neural signals during wakefulness was motivated by evidence, from fiber photometry LH recordings, that MCH neurons are activated by fear-inducing aversive events, namely electrical foot-shocks (Concetti et al., 2020). Optogenetic silencing of footshock-associated, brief MCH cell activation in awake mice produced a surprising deficiency in subsequent cued fear extinction. After acquiring fear of footshocks which were associated with auditory tones (associative fear learning), mice were exposed to fear-eliciting tones without the footshocks (safety learning), which normally leads to suppression of fear behavior (i.e., freezing in response to the tones) known as fear extinction. The optogenetic disruption of footshock-associated MCH cell activity profoundly impaired subsequent safety learning, significantly slowing down fear extinction and augmenting fear relapse (Concetti et al., 2020; Figure 4). Importantly, the MCH cell silencing in this study did not disrupt fear learning or related sensory responses, implying that MCH cell activity differentially controls safety and fear learning. While it has been early suggested that MCH neurons can modulate a number of learnt and innate behaviors as well as synaptic plasticity, no evidence previously existed that MCH cells are involved in extinction of cued fear behavior (Shimada et al., 1998; Adamantidis et al., 2005; Adamantidis and de Lecea, 2009; Guyon et al., 2009; Pachoud et al., 2010; Bittencourt, 2011; Conductier et al., 2013; Jego et al., 2013; Noble et al., 2018, 2019; Izawa et al., 2019; Kosse and Burdakov, 2019). The study of Concetti et al. (2020) proposed that MCH neurons, and specifically their activity during early stages of associative memory formation, normally serve as a neural substrate for inhibition of overactive fear behavior.

While many details of the two studies differ (Kosse and Burdakov, 2019; Concetti et al., 2020), their conceptual conclusions can be considered similar: during early “sensory experiencing” stages of memory acquisition in awake mice, the activity of MCH neurons determines whether the memory is properly behaviorally expressed later on. We do not think that these memory functions MCH neurons are in contradiction with a role in MCH neurons in forgetting recently proposed by Izawa et al. (2019). This is because the studies of Kosse et al., and Concetti et al., addressed the function of MCH cell activity waves during specific stages of wakefulness. In contrast, Izawa et al. (2019) attribute their findings to MCH cell activity during sleep, and wake and sleep – active MCH cells may be different subpopulations. In addition, Izawa et al. (2019) and Concetti et al. (2020) analyzed different aspects of fear memory, initial cued fear responses and fear extinction, respectively, and the overlapping aspects of the two paradigms produced similar results (unaltered initial cued fear responses) in the two studies.

How the transient awake signals on MCH neurons achieve these effects on memory is not yet clear. A number of cellular mechanisms have been suggested for how a transient wave of neural activity can induce a lasting transformation in the potential for future memory-related synaptic alteration, e.g., the synaptic tagging hypothesis (Redondo and Morris, 2011). At the anatomical and molecular levels, MCH cell axons and MCH receptors have been reported brain-wide, in multiple regions postulated linked to memory processing such as the hippocampus and cortex (Bittencourt, 2011; Jego et al., 2013), where MCH peptide signaling has been proposed to alter synaptic plasticity thus making memories more likely to form (Adamantidis and de Lecea, 2009; Pachoud et al., 2010). In the case of object recognition memory formation (Kosse and Burdakov, 2019), MCH cell signals may thus open “temporal windows” of synaptic state alteration during which synaptic weight changes (the cellular correlate of long-term memories) are more likely to form. In the case of fear conditioning and safety learning (Concetti et al., 2020), it is thought that during the few minutes when a new fear-association is formed the memory consolidation process is labile (McGaugh, 2000; Dudai et al., 2015). Therefore, the optogenetic disruption of MCH cells during fear conditioning might promote over-consolidation (Pitman, 1989) and strengthen the aversive memory, thus impairing extinction.

## Emerging Concepts, Unanswered Questions, and “Arousal” Arguments

The studies reviewed above, including some with direct confirmation of global brain state by EEG/EMG recordings (Mileykovskiy et al., 2005) make it clear that both orexin and MCH neurons are active outside sleep. Causal evidence is beginning to emerge that this awake activity of the LH governs fast and slow aspects of decision-making, sensorimotor control, and memory dynamics (Bracey and Burdakov, 2020; Burdakov and Peleg-Raibstein, 2020). Several key questions remain, three of which we briefly highlight here.

First, are the awake actions of orexin and MCH neurons mediated by the neuropeptides they release or by GABA/glutamate that they also co-release? This has been addressed, in some cases, by orexin/MCH receptor antagonists, where the data imply that some memory-modulating effect of MCH neurons and exploration-guiding effects of orexin neurons, indeed rely on these neuropeptides (Blomeley et al., 2018; Kosse and Burdakov, 2019). In many other cases, however, the answer is not known, for example in the case of rapid control of movement by orexin neuron dynamics (Karnani et al., 2020).

Second, are “sleep-active” and “wake-active” orexin/MCH neurons the same or different neural subpopulations? This question has been technically difficult to answer, because this requires recordings of neural ensembles across brain states. However, in the LH, this is now achievable using GRIN lenses combined with head-mounted miniscopes or 2-photon microscopy (Blanco-Centurion et al., 2019; Karnani et al., 2020). For orexin neurons, such data clearly implies that most of these cells are active during wakefulness (Karnani et al., 2020). It remains to be determined whether there is a smaller subpopulation of orexin cells that is active during sleep and in particular during sleep-state transitions. For MCH neurons, some studies indicate that a major subset of cells that are active during REM sleep are also active during awake exploratory behavior (Blanco-Centurion et al., 2019), but others argue that REM and wake active MCH cells are separate (Izawa et al., 2019). This question awaits more thorough investigation. The results of these investigations would need to be integrated with knowledge of projection targets of the LH cells, which may be distinct even within one genetically defined cell type [e.g., for orexin neurons: (Espana et al., 2005; Harris and Aston-Jones, 2006; Iyer et al., 2018), but see (Gonzalez et al., 2012)], as well as with co-transmitter and postsynaptic receptor combinations that may differ at different projection sites (Li and van den Pol, 2006; Sakurai, 2007; Baimel et al., 2017). Ultimately, it will be important to know the input-output circuits of all subpopulations of orexin and MCH neurons. In this regard, parallels to the noradrenergic neurons of the locus coeruleus might be interesting: although the projections of these cells have been originally thought to be diffuse and non-specific, recent research highlighted considerable subcircuit selectively and projection-specific postsynaptic effects (Schwarz et al., 2015; Uematsu et al., 2015; Aston-Jones and Waterhouse, 2016).

The third question, which is invariably raised at conferences and in article peer-reviews related to this topic, and relates to the title of this review, is of a more conceptual nature. It can be summarized as follows: are actions of orexin and MCH during sleep and wake fundamentally the same, and related to impact of these cells on “arousal”? For example, when orexin cells are optogenetically stimulated, do mice run more not because orexin cells are directly involved in motor control, but because mice are more awake/aroused? When MCH cells are active during object investigation, do mice then remember the object better not because MCH cells directly control fundamental molecular underpinnings of memory gating, but because MCH cells make mice more aroused and so better able to “take in” the object?

This question can be contemplated at two levels, by considering specific experimental observations, or by considering our definitions of “arousal.” To the best of our knowledge, there is little evidence that MCH neurons promote arousal, only that they promote sleep (Verret et al., 2003; Jego et al., 2013; Konadhode et al., 2013). “Increased arousal” thus seems an unlikely explanation for memory-acquisition-enhancing effects of transient MCH cell activity waves reported during novel awake experiences (Kosse and Burdakov, 2019; Concetti et al., 2020). There is evidence, however, that orexin neurons promote most aspects of arousal (Williams and Burdakov, 2008; Kuwaki, 2011; Mahler et al., 2014). However, in order to integrate the finding of rapid (subsecond) sensorimotor control by orexin neurons (Karnani et al., 2020) into their broader brain function, a scientific definition of “arousal” should be considered and agreed on. To the best of our knowledge, there is currently no scientific definition of “arousal,” and this could be a key cause of confusions and arguments in classifying neural roles as arousal or otherwise. A consensus on the operational definition of arousal in rodent studies, for example using measures such as pupillometry, is badly needed.

The classification of neural effects as “arousal effects” requires a specific temporal or anatomical definition of arousal, which seems to be largely missing from the current literature. The speed of orexin cell excitation by external stimuli can be as short as 34 ms, and orexin cell electrical excitation can cause movements as rapidly as 300 ms (Karnani et al., 2020). Consistent with this, there is evidence for direct control by orexin neurons of primary motor neurons in the spinal cord (Yamuy et al., 2004). If this still means that all actions of orexin neurons can be explained by calling them “arousal neurons,” then one may argue that neurons typically classified as sensorimotor controllers (e.g., in basal ganglia as well as motor cortex neurons) are all “arousal neurons.” Alternatively, orexin neurons can be added to the growing list of motor control neurons in the brain (Svoboda and Li, 2018). Indeed, there are great similarities between motor roles of orexin and dopamine systems. For example, da Silva et al., find that midbrain dopamine neurons mediate self-initiated locomotion, and have a variety of activity profiles during action initiation, with some neurons turning off and some on, i.e., the same as observed for orexin cells (da Silva et al., 2018; Karnani et al., 2020). Like orexin neurons, the striatal movement-control neurons activate before self-initiated actions (Cui et al., 2013). A coherent conceptual progress in this field may need a modernization of terminology referring to different timescales at which brain signals are coupled to behavior.

## Author Contributions

Both authors listed have made a substantial, direct and intellectual contribution to the work, and approved it for publication.

## Conflict of Interest

The authors declare that the research was conducted in the absence of any commercial or financial relationships that could be construed as a potential conflict of interest. The reviewer TS declared a past co-authorship with one of the authors DB to the handling Editor.
